# Machine learning approaches to cryoEM density modification differentially affect biomacromolecule and ligand density quality

**DOI:** 10.3389/fmolb.2024.1404885

**Published:** 2024-04-18

**Authors:** Raymond F. Berkeley, Brian D. Cook, Mark A. Herzik

**Affiliations:** Department of Chemistry and Biochemistry, University of California San Diego, La Jolla, CA, United States

**Keywords:** cryogenic electron microscopy, cryoEM, density modification, model building, machine learning

## Abstract

The application of machine learning to cryogenic electron microscopy (cryoEM) data analysis has added a valuable set of tools to the cryoEM data processing pipeline. As these tools become more accessible and widely available, the implications of their use should be assessed. We noticed that machine learning map modification tools can have differential effects on cryoEM densities. In this perspective, we evaluate these effects to show that machine learning tools generally improve densities for biomacromolecules while generating unpredictable results for ligands. This unpredictable behavior manifests both in quantitative metrics of map quality and in qualitative investigations of modified maps. The results presented here highlight the power and potential of machine learning tools in cryoEM, while also illustrating some of the risks of their unexamined use.

## 1 Introduction

Over the past decade, cryogenic electron microscopy (cryoEM) has matured into a leading method for determining the three-dimensional (3-D) structures of dynamic macromolecular complexes (DiIorio and Kulczyk, 2022). Reductions in the cost of GPU-accelerated computing coupled with improvements in both microscope and detector hardware have facilitated the acquisition of high-quality, high-resolution cryoEM data (Nakane et al., 2020; Yip et al., 2020; Fréchin et al., 2023). Hardware improvements have been complemented by concurrent developments in cryoEM data processing software, drastically improving both the performance and accessibility of the cryoEM data processing pipeline (Scheres, 2012; Punjani et al., 2017; Tegunov and Cramer, 2019). These efforts have enabled the democratization of cryoEM, allowing researchers with a range of scientific backgrounds to determine high-resolution structures of diverse biomacromolecules using cryoEM (Baldwin et al., 2018).

The same cost reductions and performance improvements in computational hardware that have driven advances in cryoEM have also bolstered advances in the application of machine learning (ML) methodologies at nearly all stages of the cryoEM workflow. ML has been applied to cryoEM data particle identification and curation, 3-D classification and reconstruction, and map modification (Bepler et al., 2019; Tegunov and Cramer, 2019; Wagner et al., 2019; Bepler et al., 2020; Sanchez-Garcia et al., 2021; Zhong et al., 2021; Chung et al., 2022; Kimanius et al., 2023; Punjani and Fleet, 2023). Map modification approaches in particular have benefitted substantially from the application of machine learning, with ML-based methods providing the ability to encode information about cryoEM maps that are difficult to capture using previous map modification methods like *B*-factor sharpening. This deep encoding often leads to a high-quality EM map that is more featureful and interpretable than a map generated by traditional map modification or sharpening approaches alone.

While ML-based map modification approaches have demonstrated the ability to enhance features within cryoEM maps, they also have the potential to introduce artifacts or bias into maps that could negatively impact the quality and interpretation of the EM density. As ML-based map modification approaches become a standard feature of cryoEM data processing workflows, it will be important to understand and mitigate any biases that could alter densities in a way that meaningfully alters the scientific conclusions from an experiment. The growing reliance on structure determination workflows that simultaneously incorporate automated ML-based protein structure prediction (Jumper et al., 2021), map modification (Sanchez-Garcia et al., 2021; He et al., 2023; Maddhuri Venkata Subramaniya et al., 2023), and model building (Jamali et al., 2024), make this need even more pressing. To highlight some of the nuance that is inherent in the application of ML-based methods in cryoEM, this perspective aims to demonstrate a handful of examples of the application of traditional, non-traditional, and ML-based cryoEM map modification approaches and highlight some of their benefits and pitfalls. Particular emphasis in this manuscript is directed towards non-protein molecules within EM densities, which we find are particularly affected by these tools in unpredictable ways.

### 1.1 Map modification by degrees

Post-reconstruction processing of cryoEM maps (hereafter referred to as “map modification”) can be a critical step at the end of the cryoEM data processing pipeline, where it can aid in model building and reveal features in maps that would have not been visible or obvious otherwise. Map modification approaches typically rely on the integration of prior knowledge about the distribution or nature of features in cryoEM maps that are attenuated by the cryoEM data acquisition and reconstruction processes. The degree to which prior knowledge is incorporated can range from simple corrections based on assumptions about the ratio of low-to high-spatial frequency information in the data, to the wholesale voxel-by-voxel modification of the map by a neural network. Interpretation of a modified cryoEM map must be performed within the context of the potential bias that is introduced by the map modification approach that was used to generate it.

The most widespread map modification approach is *B*-factor sharpening, which aims to correct for the inherent loss of contrast observed at high spatial frequencies in experimental cryoEM maps by adjusting map signal amplitudes within a certain frequency range. (Rosenthal and Henderson, 2003; Fernandez et al., 2008). Historically, this correction has been performed to all regions of the map simultaneously by measuring the relative loss of high spatial frequency components in the data and dampening signal at low spatial frequencies to restore the ratio of low and high frequency components in the data. This global sharpening approach benefits from being simple and grounded in a straightforward interpretation of the data, and, for these reasons, is the default sharpening approach implemented in cryoEM data processing packages, such as RELION and cryoSPARC (Scheres, 2015; Punjani et al., 2017).

While global *B*-factor sharpening is effective, simple, and even tunable by the cryoEM practitioner, there is still room for improvement: global sharpening is susceptible to local over- or under-sharpening that results from the heterogeneous attenuation of high spatial frequency information in different regions of the map. Recently developed sharpening algorithms incorporate additional information into the signal weighting strategy in order to achieve local, rather than global sharpening (Jakobi et al., 2017; Ramírez-Aportela et al., 2019; 2020; Kaur et al., 2021). One method to facilitate local sharpening is to incorporate information about the local map resolution (Ramírez-Aportela et al., 2020). Another approach is to use information about the model to guide signal weighting across the map. Since the degree of low frequency signal attenuation is dependent on the chemistry and structure of the molecule being imaged, knowledge about the model can then be used to enhance local map quality through model-based density sharpening (Jakobi et al., 2017). While these methods improve local sharpening performance, this improvement comes at the cost of bias, either from the model or from assumptions embedded in local resolution estimates. Newer approaches that leverage spiral phase transformations to achieve local sharpening show promise for a less biased future in this area (Kaur et al., 2021).

Further post-processing approaches exist beyond global and local sharpening. One such post-processing approach is the resolve_cryo_em algorithm in PHENIX (hereafter referred to as PHENIX RESOLVE), which implements an algorithm that uses a maximum-likelihood approach to estimate and correct uncorrelated errors in two-half maps (sharpened or unsharpened) following a gold-standard refinement. This error correction coincides with map sharpening and results in a comprehensive map modification approach that only relies on the assumptions that amplitude and phase errors in the input half maps are Gaussian and that the solvent region of the map only contains uniform noise (Terwilliger et al., 2020). This error correction approach can often improve the quality of the map more than sharpening, which makes PHENIX RESOLVE a useful tool for map modification.

Machine learning has opened new avenues to map modification beyond the traditional approaches described above. Just like map sharpening attempts to improve map quality by adjusting the amplitude of signal in the map to reproduce ratios of low to high frequency signal expected in proteins, ML-based map modification algorithms aim to introduce or enhance features that would be expected to be present in a high-quality cryoEM map. This encoding is embedded in a trained model that can be used to inject these map-like features into a raw map. Unlike sharpening, many of the tools that leverage ML to accomplish map modification operate on volumetric data directly. Examples of ML tools in this class are DeepEMHancer (Sanchez-Garcia et al., 2021), EMReady (He et al., 2023), and EM-GAN (Maddhuri Venkata Subramaniya et al., 2023).

While ML-based map modification tools can dramatically improve the quality and interpretability of a map, they also have the potential to introduce biases that could not be drawn from simple manipulations of the frequency domain of the map. Map composition is imbalanced: since most of the useable signal in a given map represents protein, protein-like features come to dominate the resulting models, with less emphasis on density representing bound nucleic acids, lipids, small molecules, cofactors, or post-translational modifications. A similar representation problem arises when using synthetic maps as training targets, since many structures in the PDB are incompletely modeled or do not contain ligands, and therefore generate synthetic target maps that are ligand-free (Sanchez-Garcia et al., 2021). It follows that these biases may be incorporated into ML models that generate modified maps that cause a user to miss non-protein map components, especially if there is no prior knowledge that the target density contains these components.

## 2 ML-based map modification aids in map interpretation and generally improves model-map fit statistics

To systematically evaluate the effect of bias in ML-based cryoEM map modification approaches, we compared the performance of three ML-based map modification methods–DeepEMHancer, EMReady, and EM-GAN–with traditional B-factor-based map sharpening using deposited maps in the EMDB. We also evaluated PHENIX RESOLVE to provide a point of comparison as a map modification approach that is more fully-featured than *B*-factor sharpening but does not use ML. We applied each of these tools to a collection of cryoEM maps containing non-protein elements such as ligands, cofactors, metals, nucleic acids, and lipids, to assess how each method handles these components against the protein background. Included in this collection is alcohol dehydrogenase, which has multiple zinc and nicotinamide adenine dinucleotide (NAD) cofactors (EMD-0406, 2.9 Å) (Herzik Jr et al., 2019); β-galactosidase, a structure containing both zinc and magnesium cofactors and a small molecule phenylethyl β-D-thiogalactopyranoside ligand (EMD-7770, 1.9 Å) (Bartesaghi et al., 2018); T20S proteasome, a common benchmark protein for cryoEM methods development (EMD-8741, 3.1 Å) (Herzik Jr et al., 2017); a structure of the centromeric nucleosome, including the surrounding DNA (EMD-8949, 2.6 Å) (Zhou et al., 2019); connexin-46/50, a membrane protein whose structure is stabilized by lipids at cell junctions (EMD-22358, 1.9 Å) (Flores et al., 2020); Na_v_1.7, solved in complex with a clinically-relevant small molecule antagonist (EMD-35193, 2.7 Å) (Wu et al., 2023); and hemoglobin in complex with oxygen (unpublished, 2.4 Å). These maps were selected due to their quality, high resolution, and the variety of cofactors/ligands/ions that they contain alongside protein and nucleic acid biomacromolecules.

To provide a quantitative overview of the performance of the map modification techniques in our panel, we calculated the Q-scores for each map in our dataset. Q-scores serve as a quantitative proxy for the atom’s resolvability within the map and the quality of the model-map fit by measuring the fit of the map density around atoms in an atomic model (Pintilie et al., 2020). Q-scores were calculated using models that had been subjected to PHENIX real-space refinement into their respective modified map in order to optimize the fit of the model into each modified map and thereby provide a more balanced comparison of map quality (Afonine et al., 2018).

**FIGURE 1:**
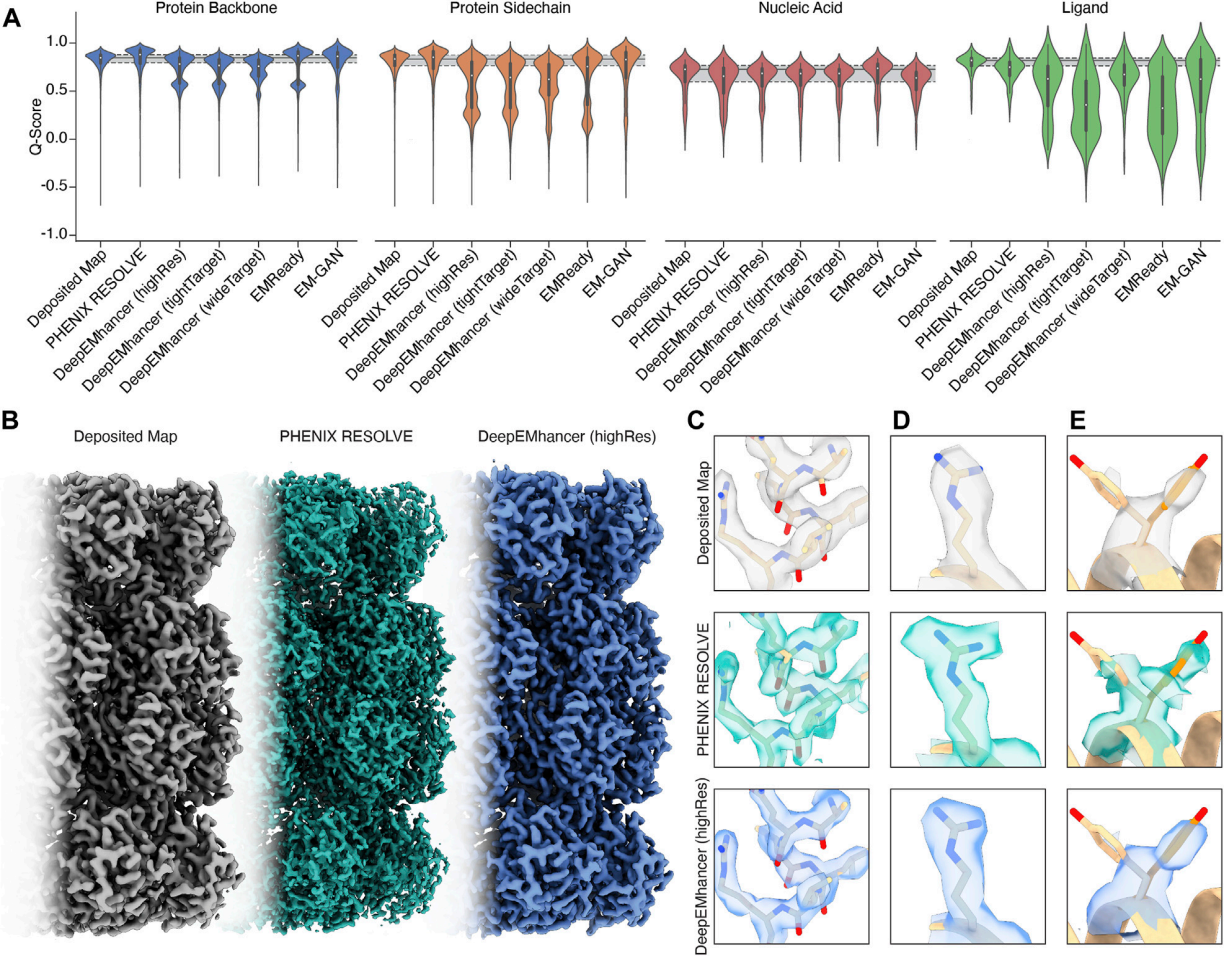
An overview of the performance of map modification approaches. **(A)** Aggregate Q-scores of backbone, sidechain, nucleic acid, and ligand density in our dataset. The mean and interquartile ranges for the Q-scores of the deposited map are represented by gray bars that extend across the violin plots for each density type. **(B)** *Thermoplasma acidophilum* 20S proteasome density before (left) and after map modification by PHENIX RESOLVE (middle) and DeepEMhancer (right). **(C)** An example of protein backbone density before (top) and after map modification by PHENIX RESOLVE (middle) and DeepEMhancer (bottom). **(D)** An example of protein sidechain density before (top) and after map modification by PHENIX RESOLVE (middle) and DeepEMhancer (bottom). **(E)** An example of heterogeneous rotamer conformations before (top) and after map modification by PHENIX RESOLVE (middle) and DeepEMhancer (bottom).

For regions of our maps representing protein, processing by PHENIX RESOLVE, EMReady, and EM-GAN generally improved Q-scores beyond those observed in deposited maps (Figure 1A), indicating that these approaches yielded EM densities that allowed for a better map-model fit after real-space refinement using PHENIX. All three DeepEMhancer models slightly reduced calculated Q-scores. Because DeepEMhancer provides multiple models to evaluate maps generated with different masking approaches, this reduction in the Q-score distribution could be partially attributed to mismatches between the specific DeepEMhancer model (highRes, tightTarget, or wideTarget) used and the masks on each of the volumes in our evaluation set. Since all the maps in our panel exhibit sub-4 Å resolution, however, it is likely that the highRes model is appropriate for all maps. It is possible that poor performance by DeepEMhancer on EMD-22358, which accounts for the majority of the Q-scores represented in the lower mode of the bimodal density evident in the violin plots for DeepEMhancer results on the protein backbone, is skewing the distribution for DeepEMhancer. Overall, most ML-based map modification improves Q-scores of protein density in maps, but the extent varies across the approaches.

To further evaluate the effect of the post-processing approaches in our panel on protein densities, we wanted to individually assess each of our test cases in detail. For the *Thermoplasma acidophilum* 20S proteasome (T20S) (Herzik Jr et al., 2017)PHENIX RESOLVE generated more featureful maps, while ML-based methods tended to generate smoother maps with clearer backbone density (Figures 1B,C).As expected based on our Q-score analysis, details in the protein backbone were enhanced by many of the map modification techniques that we evaluated. In particular, backbone carbonyl densities that were weak or absent in the *B*-factor-sharpened maps were much clearer and more pronounced following modification with PHENIX RESOLVE, and to a lesser degree DeepEMhancer, EMReady, and EM-GAN. Importantly, we speculate these enhancements in features facilitated better orientation of the backbone carbonyl for more accurate modelling of the protein backbone (Figure 1C), which is in agreement with our observation that map modification and subsequent model refinement generally conferred a slight improvement in backbone Q-scores (Figure 1A) and Ramachandran statistics. In addition to improvements in backbone modeling, map modification also typically enhanced the quality of the density around most sidechains, with improvements in the shape and fit for preexisting densities throughout the maps that we evaluated (Figure 1D). Qualitative improvements in sidechain densities were validated with improvements in Q-scores, with PHENIX RESOLVE performing the best overall and DeepEMHancer yielding inconsistent results, depending on the map. Surprisingly, there did not appear to be a correlation in rotamer outlier statistics after map modification of any kind.

Beyond improvements in backbone and sidechain densities, interpretability improvements extend to ambiguities in the map which may have otherwise confounded model building. As an example, Tyr 58 in the β-subunit of T20S has been shown to adopt two preferred rotamer conformations (Figure 1E) (Herzik Jr et al., 2017). In the *B*-factor-sharpened map, the density corresponding to the more populated rotamer is visible with an occupancy of 53%. The less populated rotamer, despite an occupancy of 47%, could easily be missed in the model building process, especially if automated model-building strategies were utilized. The application of PHENIX RESOLVE adjusts the density of the less populated rotamer enough to investigate it during model building, with a clear branch in the density corresponding to each rotamer. Features in the densities of both rotamers that are not visible in the *B*-factor-sharpened map appear after density modification by PHENIX RESOLVE. The application of any of the three ML-based map modification approaches, on the other hand, affects the density in the opposite way by enhancing the density of the more populated rotamer, with an average occupancy of 67% for the more populated rotamer and 32% for the less populated rotamer. The value of this modification depends on the goal of the map modification experiment–while it does improve the “protein-likeness” and potential interpretability of the map by improving the density for the more populated rotamer, the ability to identify and place the minor rotamer does not improve in comparison to the *B*-factor-sharpened map. This observation demonstrates the potential value of map modification beyond improvements in modeling, as these approaches can lead to the discovery of distinct entities in the map, but underscores the importance of comparing the performance of various map modification approaches on the same map.

The Q-score distributions for nucleic acids were relatively unaffected by any of the map modification approaches in our panel, except for EMReady, which was the only method in our evaluation that conferred an overall Q-score improvement to nucleic acids (Figure 1A). The relatively unchanged Q-score distributions belie differences in the modification results for the DNA backbone and nucleobase densities which are apparent upon a closer, qualitative inspection of the map that will be discussed in the following section. In general, biases in ML-based map modification approaches do not seem to be pronounced for nucleic acids, and our evaluation suggests that the application of map modification methods is warranted for regions of EM maps representing nucleic acid. Perhaps in future implementations, greater populations of nucleic acid-containing EM maps will be incorporated into the training sets.

## 3 Ligand densities are disproportionately affected by ML-based map modification

Unlike the quality enhancements observed for regions of the maps representing protein, ligand densities were almost always negatively impacted by the map modification approaches in our test panel. This impact was exaggerated for ML-based map modification approaches, with the distribution of Q-scores for all ML-based methods being significantly worse than those for the deposited sharpened maps and the PHENIX RESOLVE maps. Densities representing ordered water molecules were also disproportionately impacted by ML-based map modification approaches (Figure 1A). Our dataset includes structures that harbor an array of cofactors and ligands and thus the trends we observe can be generalized broadly to cofactors and ligands not explicitly investigated here.

**FIGURE 2:**
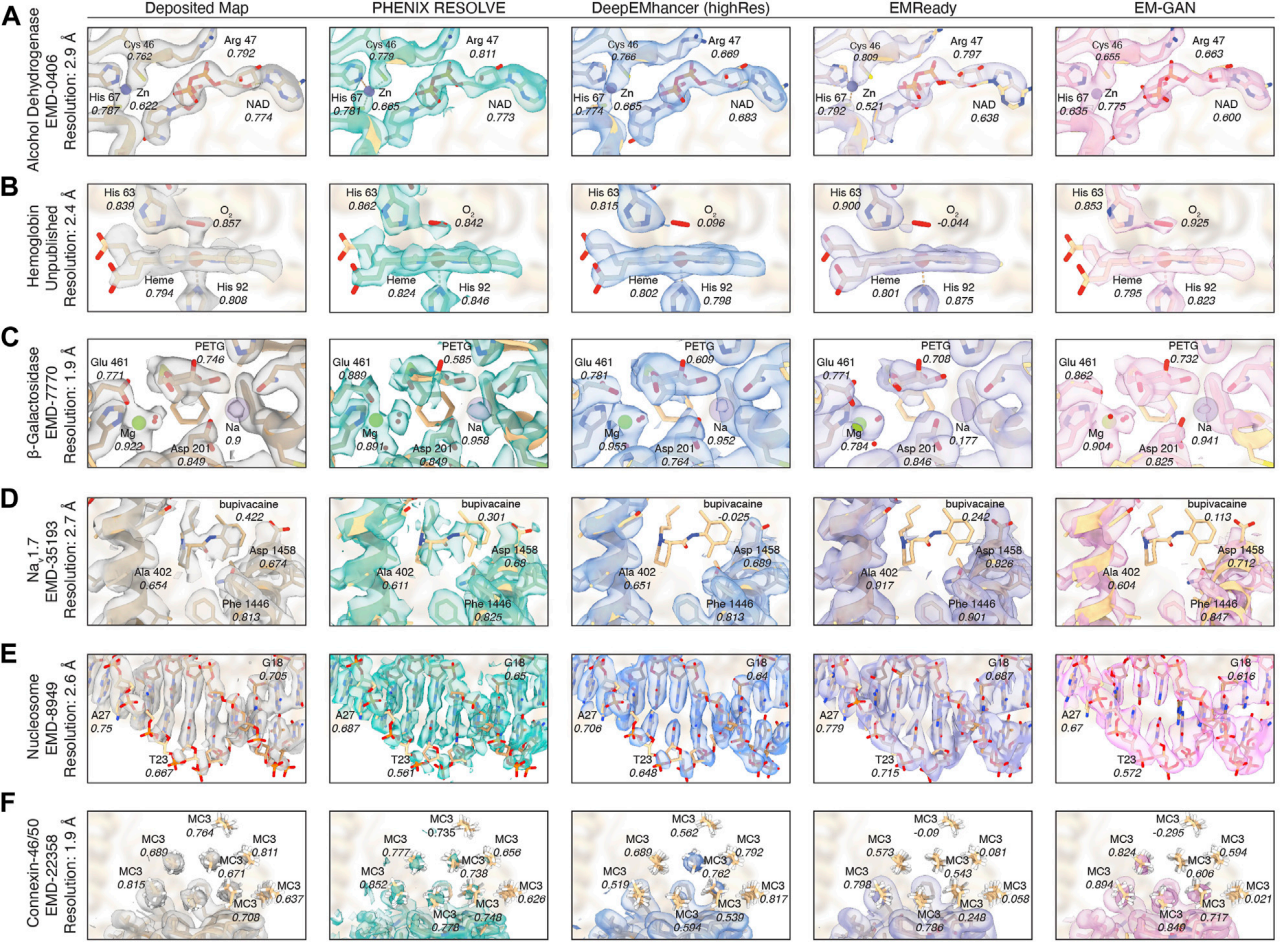
Ligand densities in focus after map modification approaches. EM density before and after modification for **(A)** NAD and Zn^2+^ densities in the active site of alcohol dehydrogenase. **(B)** Heme and O_2_ densities in hemoglobin. **(C)** PETG, Mg^2+^, and Na^+^ densities in the active site of β-galactosidase. **(D)** Bupivacaine in the active site of Na_v_1.7. **(E)** Nucleic acid densities in a centromeric nucleosome. **(F)** Lipid densities surrounding the structure of connexin-46/50.

In light of the performance of ML-based map modification approaches on ligands and waters in our Q-score statistics, we sought to further evaluate affected regions of the maps in our evaluation set on an individual basis to gauge map quality near ligand sites. We first chose to investigate the impact of map modification on the 2.9 Å EM map for alcohol dehydrogenase (EMD-0406) (Herzik Jr et al., 2019), which contains both catalytic and structural metal sites, as well as a bound NAD cofactor. The catalytic zinc cofactor is coordinated by Cys 46, His 67, and Cys 174 in the active site pocket of each subunit. In the deposited map, the zinc can be resolved, and the refined model has a Q-score of 0.622. All of the map modification tools that we examined performed acceptably with the zinc cofactor in this system–the Q-score is not significantly affected by any of the map modification approaches. DeepEMhancer and EM-GAN both yield a qualitative improvement in the active site density in addition to an improved Q-score. EMReady appears to remove a significant portion of the zinc density but confers a significant improvement to the map quality for each of the residues in complex with the zinc cofactor, an effect that would likely enable more accurate model building even if the zinc density itself is diminished. (Figure 2A). On the contrary, the NAD cofactor density was either maintained or was made more featureful using the modification approaches in our panel, and we speculate, due to their similarity in composition, the observed effects on nucleic acids in the nucleosome map parallel here.

We next evaluated a 2.4 Å EM map of human hemoglobin, which contains four heme cofactors, each bound to a molecule of O_2_. In our evaluation, the densities for the heme cofactors remained relatively unaffected by the post-processing approaches in our panel. Qualitatively, the density was improved the most by PHENIX RESOLVE, and all methods conferred a small improvement in the Q-score. The O_2_ ligand, on the other hand, did not fare well in our evaluation, as it was completely removed by both DeepEMhancer and EMReady (Figure 2B). Given the significant interest in the ligand for its structural and functional roles in hemoglobin, its absence in the density map severely hinders accurate structural characterization of the protein. Its removal provides an example of the unpredictable nature of ligand handling by map modification approaches–it is not immediately clear why the O_2_ density would be removed by two of the four methods in our panel while being unaffected by the other two.

To investigate the performance of the tools in our map modification panel on a small molecule ligand, we next investigated the 1.9 Å map of β-galactosidase bound to the small molecule β-galactosidase inhibitor, phenylethyl β-D-thiogalactopyranoside (PETG) (EMD-7770) (Bartesaghi et al., 2018). The density for PETG is retained by each of the methods in our panel, with only slight reductions in Q-score, although the density for the phenylethyl moiety was altered differently across the panel. In addition to the ligand, the active site of β-galactosidase contains magnesium and sodium ions along with multiple structural waters, all of which are resolved and have high Q-scores in the deposited map. Surprisingly, despite the retention of the PETG ligand density in each of the modified maps, the metals were disproportionately affected, despite the high resolution of all cofactors and ligands in this map. The density for the magnesium ion and coordinated waters were enhanced in the PHENIX RESOLVE and EM-GAN maps but with less featureful densities observed in the DeepEMhancer and EMReady maps. Notably, the sodium cofactor is completely removed from the map after map modification by EMReady and weakened in the DeepEMhancer and EM-GAN maps (Figure 2C).

To further evaluate small molecule ligands, we turned to the 2.7 Å resolution structure of Na_V_1.7 in complex with the agonist bupivacaine (EMD-35193) (Wu et al., 2023). The clinical relevance of this system offers a compelling example of a small-molecule ligand for which high-resolution structural information could provide valuable insights for structure-based drug design and medicinal chemistry efforts. For this system, all ML-based map modification approaches failed–removing the bupivacaine EM density in its binding site in each case (Figure 2D). Even though the interpretability and Q-scores of side chain densities improved, particularly in the EMReady map, the removal of the bupivacaine density would compromise any analysis of its orientation and geometry bound to Na_V_1.7. ML-based map modification in this scenario would therefore cripple the interpretation of bupivacaine-Nav1.7 interactions in the ML-modified maps, even if the protein density statistics are improved.

We next sought to determine the impact of map modification on regions of a cryoEM map representing nucleic acid. To this end, we evaluated a map of the centromeric nucleosome (EMD-8949) (Zhou et al., 2019). Notably, unlike most nucleosome structures, which are solved in complex with the stable Widom 601 DNA sequence (Lowary and Widom, 1998), the nucleosome in this map is wrapped with a 145 base pair α-satellite DNA sequence (Zhou et al., 2019). The novelty of this sequence is valuable given the likely enrichment of Widom 601 structures present in the training data used by the ML-based map modification approaches in our panel. As suggested by the minimal impact of map modification on the Q-score distributions for the nucleic acid densities (Figure 1A), the map modification approaches in our panel generally perform well on the nucleic acid portion of this map. Map modification seems to have a non-uniform effect on nucleic acid densities, with most methods improving the resolvability of the backbone and pentose and eroding density for the nucleobase. Even with this effect, the interpretability improvements afforded by improvements in the resolvability of the DNA backbone suggest that map modification can be valuable for interpreting cryoEM maps of nucleic acid.

Finally, we investigated the impact of map modification on lipids using the 1.9 Å map for connexin-46/50 (EMD-22358) (Flores et al., 2020), a channel protein involved in the formation and maintenance of intercellular gap junctions. The map contains a series of well-resolved annular lipids that are known to stabilize the structure of connexin-46/50 (Locke and Harris, 2009). The lipids are arranged in a lattice that extends radially from the cylindrical transmembrane protein structure. Interestingly, while no map modification approaches conferred an overall improvement to lipid densities, both EMReady and EM-GAN affected the Q-scores of the lipid densities significantly by enhancing the resolution of lipids near the protein while almost completely removing lipid densities further away from the protein (Figure 2E).

In summary, we provide a selection of densities from the maps in our evaluation before and after map modification by the approaches in our panel. This selection supports our initial observations that map modification tends to improve the quality and interpretability of biomacromolecule densities in cryoEM maps. A closer look a selection of ligands suggests that the performance of map modification is more sporadic for the ligands themselves, with ML-based map modification more often introducing extreme results that frequently removed selected ligands from maps entirely.

## 4 Discussion

Map modification is a crucial step in the cryoEM data processing and model building pipeline. ML-based map modification techniques provide an orthogonal approach to map modification approaches that has the potential to provide insight into details in cryoEM maps that may not be captured with traditional map sharpening and map modification approaches like PHENIX RESOLVE. Although ML-based methods have great potential for revealing attenuated features in cryoEM data, the black-box nature of neural networks and the potential for bias inherent in these techniques means that their application requires awareness of their potential pitfalls. In this perspective, we provide an evaluation of map modification tools to present the benefits and limitations of these techniques, with a focus on the performance contrast between traditional and ML-based map modification methods. We show that while map modification by any of the popular ML-based methods often improves map quality and interpretability, the improvement can come at the cost of map quality for density in the map that does not represent protein. In our analyses, these missing densities included structural ions and metals whose geometries can provide crucial insight into protein structure and function, and clinically relevant small molecules whose binding poses can inform medicinal chemistry efforts. The conclusion is that while ML-based volume postprocessing approaches can be extremely powerful, their use must be performed with the understanding that real non-protein density could be negatively impacted or even removed from cryoEM maps. We therefore recommend that ML-based density modification approaches should be used to complement, rather than replace, traditional map modification approaches combined with careful analysis of the unmodified map. We hope that this evaluation will encourage the use of ML-based volume post processing approaches when combined with careful consideration of complementary *B*-factor-sharpened maps.

## Data Availability

The data presented in the study are deposited in Zenodo, accession number 10934245, doi: 10.5281/zenodo.10934245.
